# Role of the GRAS transcription factor ATA/RAM1 in the transcriptional reprogramming of arbuscular mycorrhiza in *Petunia hybrida*

**DOI:** 10.1186/s12864-017-3988-8

**Published:** 2017-08-08

**Authors:** Mélanie K. Rich, Pierre-Emmanuel Courty, Christophe Roux, Didier Reinhardt

**Affiliations:** 1 Department of Biology, University of Fribourg, Rte Albert-Gockel 3, 1700 Fribourg, Switzerland; 2 Laboratoire de Recherche en Sciences Végétales, Université de Toulouse, CNRS, UPS, 24 Chemin de Borde Rouge-Auzeville, 31326 Castanet-Tolosan, France; 3 Present address: Agroécologie, AgroSupDijon, CNRS, INRA, Université de Bourgogne Franche-Comté, 21000 Dijon, France

**Keywords:** *Petunia hybrida*, Arbuscular mycorrhiza, Symbiosis, RAM1, GRAS transcription factor

## Abstract

**Background:**

Development of arbuscular mycorrhiza (AM) requires a fundamental reprogramming of root cells for symbiosis. This involves the induction of hundreds of genes in the host. A recently identified GRAS-type transcription factor in *Petunia hybrida*, ATA/RAM1, is required for the induction of host genes during AM, and for morphogenesis of the fungal endosymbiont. To better understand the role of RAM1 in symbiosis, we set out to identify all genes that depend on activation by RAM1 in mycorrhizal roots.

**Results:**

We have carried out a transcript profiling experiment by RNAseq of mycorrhizal plants vs. non-mycorrhizal controls in wild type and *ram1* mutants. The results show that the expression of early genes required for AM, such as the strigolactone biosynthetic genes and the common symbiosis signalling genes, is independent of RAM1. In contrast, genes that are involved at later stages of symbiosis, for example for nutrient exchange in cortex cells, require RAM1 for induction. RAM1 itself is highly induced in mycorrhizal roots together with many other transcription factors, in particular GRAS proteins.

**Conclusion:**

Since *RAM1* has previously been shown to be directly activated by the common symbiosis signalling pathway through CYCLOPS, we conclude that it acts as an early transcriptional switch that induces many AM-related genes, among them genes that are essential for the development of arbuscules, such as STR, STR2, RAM2, and PT4, besides hundreds of additional RAM1-dependent genes the role of which in symbiosis remains to be explored. Taken together, these results indicate that the defect in the morphogenesis of the fungal arbuscules in *ram1* mutants may be an indirect consequence of functional defects in the host, which interfere with nutrient exchange and possibly other functions on which the fungus depends.

**Electronic supplementary material:**

The online version of this article (doi:10.1186/s12864-017-3988-8) contains supplementary material, which is available to authorized users.

## Background

Arbuscular mycorrhiza (AM) represents a mutualistic interaction between most land plants and fungi (Glomeromycota) that culminates in the exchange of nutrients between the two partners. For the plant, the main benefit of AM is the increased supply with essential nutrient elements such as phosphorus (P), nitrogen (N), sulfur (S), copper (Cu), and zinc (Zn) [1, 2]. The functional changes associated with AM development require fundamental reprogramming of root cells, to allow them to be colonized by AM fungi and to generate the symbiotic machinery required for long-term compatibility and nutrient exchange. The establishment of AM proceeds through several stages, i.) the exchange of diffusible signals, ii.) mutual recognition, iii.) symbiotic signaling to the nucleus in cells of the host iv.) induction of AM-related genes in the host, v.) cellular rearrangement that allows accommodation of the AM fungus, and, vi.) construction of the symbiotic interface (reviewed in [3–5]).

The development of AM has been characterized in considerable detail in several AM host model species, in particular *Medicago truncatula*, *Lotus japonicus*, rice (*Oryza sativa*), and petunia (*Petunia hybrida*). The first presymbiotic signal is the constitutively released root-born phytohormone strigolactone (SL), which triggers hyphal branching and induces mitochondrial metabolism in AM fungi [6–9]. SL biosynthesis has been well characterized in petunia, pea (*Pisum sativum*), *Arabidopsis thaliana* and rice [10], and an SL transporter has been identified in petunia, which promotes AM development [11]. AM fungi, in turn, release chitin-derived signal molecules (myc factors) [12, 13] that are thought to be recognized in the plant host by lysin motif (LysM) receptor like kinases, in analogy to nod factor receptors in the root nodule symbiosis (RNS) of the legumes [14].

Upon recognition of these symbiotic signals, a signaling cascade is triggered which is required for both AM and RNS, and therefore is referred to as the common symbiosis signaling pathway (CSSP). The CSSP involves the symbiosis receptor-like kinase SYMRK at the plasma membrane [14], and a characteristic repetitive calcium transient in and around the nucleus (calcium spiking) [15]. This calcium signal is perceived and integrated by a dedicated nuclear calcium- and calmodulin-dependent protein kinase (CCaMK) [16]. CCaMK interacts with, and activates, the transcription factor (TF) CYCLOPS (CYC) [17], which triggers a transcriptional cascade resulting in the induction of hundreds of genes that are required for the establishment of the symbiotic interaction.

Induction of symbiotic gene expression during AM has been studied in several host model species such as *M. truncatula*, rice, petunia, *L. japonicus*, and tomato (*Solanum lycopersicum*) [18–24]. These studies revealed that a set of conserved genes is induced in mycorrhizal roots, indicative of a conserved AM-related transcriptional program in different host species [18]. The best-studied example of such a conserved gene encodes an AM-specific phosphate transporter (PT), represented by StPT3 in potato (*Solanum tuberosum*) [25], PhPT4 in petunia [26], MtPT4 in *M. truncatula* [27], LjPT3 in *L. japonicus* [28], PtPT11 in *Populus trichocarpa* [29], OsPT11 in rice [30, 31], and SbPT11 in *Sorghum bicolor* [32]. A large group of AM-induced or AM-specific genes encode proteases [33], the function of which remains poorly understood.

Since hundreds of genes have to be coordinately activated in mycorrhizal roots, TFs can be expected to play a critical role in AM-related gene induction. Two TFs have been characterized in considerable detail, CYCLOPS (CYC) [17], and RAM1 [34–37]. CYC directly interacts with CCaMK, and therefore may act as one of the earliest AM-related TFs [17]. One of the functions of CYC is to activate RAM1 by direct binding to the RAM1 promoter at a conserved palindromic promoter element known as the GGCGCC box [36]. Since AM development in *cyc* mutants can be restored by overexpression of RAM1 [36], it appears that RAM1 is downstream of CYC, and that CYC acts mainly or exclusively through RAM1.

While an earlier study proposed that RAM1 may act very early in presymbiotic signaling [34], recent reports suggest that its role may rather be late in intracellular accommodation of the fungal partner in host cells, and in the establishment of the symbiotic interface during the formation of the highly branched arbuscules [35–37]. Consistent with this idea, *RAM1* expression is highly induced in mycorrhizal roots [34–37].

To reveal at which level of the AM interaction RAM1 acts, an RNAseq experiment was performed on mycorrhizal roots of wild type *P. hybrida*, and in *ram1* mutants [37], using a nurse plant inoculation system to force fungal infection in both genotypes. We then explored by comparative transcriptome profiling the effect of the *ram1* mutation on the expression of groups of genes that are known to act at distinct stages early or late during AM development. In particular, we evaluated the expression of SL-related genes, CSSP components (common SYM genes), infection-related genes, and genes related to arbuscule functioning [3].

Our results show that the genes involved at early stages (SL-related genes and components of the CSSP) are not affected by the *ram1* mutation. In contrast, most AM-induced genes exhibited strongly reduced levels of expression in *ram1* mutants. In particular genes involved in arbuscule functioning, for example *PT4* and *STR*, are strongly dependent on *RAM1*. Genes involved in infection take an intermediate position, since they are partially affected by *ram1* mutations.

An interesting outcome of our study is the finding that a large number of GRAS genes are induced in mycorrhizal roots. In particular, a dicot-specific clade that was recently described as the MIG clade [38] is highly induced in mycorrhizal roots.

## Results

### Experimental design

**Fig. 1:**
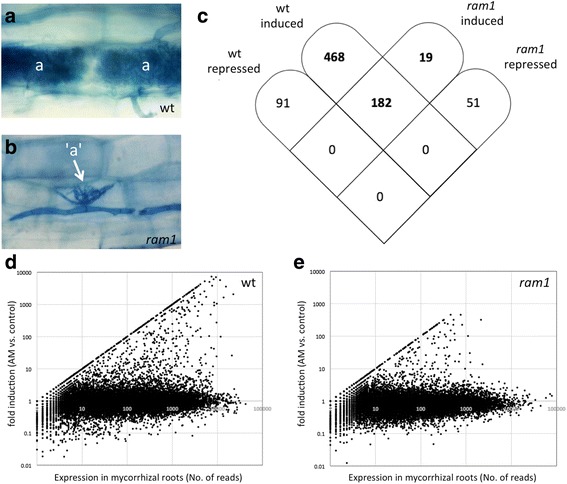
Global gene expression pattern in wild type petunia and in *ram1* mutants. **a**, **b** Arbuscular (*) and arbuscule-stunted (*arrow*) phenotypes of wild type (**a**) and *ram1* mutant (**b**), respectively. **c** Comparison of the transcriptional changes in petunia wildtype and *ram1* mutants. For both genotypes, gene expression in mycorrhizal roots was compared to non-mycorrhizal control roots. Only genes with a significant change of at least 5-fold induction or repression are shown. **d**, **e** Global gene regulation pattern in wild type (**d**), and *ram1* (**e**). Each point represents a gene and its regulation in mycorrhizal roots versus controls. For AM-specific genes, the control expression was set to 1, therefore, they appear as an increasing straight line of points (number of reads = fold induction)

Mutations in the transcription factor gene *RAM1* of *P. hybrida* result in abortion of AM [37], in particular of arbuscule formation (Fig. 1a, b). Since a transcription factor in the host is unlikely to act directly on the AM fungus, we reasoned that the mutant phenotype could be explained by defects in the induction of essential symbiosis-related genes in the plant. In order to explore the global transcriptional changes associated with AM symbiosis in *P. hybrida*, and to identify genes of the host the expression of which depends on *RAM1*, an RNAseq analysis was performed with petunia wild type and *ram1-2* mutants (further referred to as *ram1*). To achieve comparable mycorrhizal colonization levels in wild type and the AM-defective *ram1* mutant [37], nurse plant inoculation was used, which results in wild type levels of overall colonization in mutants, although the qualitative aspects of the mutant phenotype in symbiotic development are retained [37, 39]. Mycorrhizal roots of three biological replicate plants with an average colonization level of 20% ± 5% and 15% ± 8% in wild type and *ram1*, respectively, were used along with three non-colonized controls. In each treatment, ca. 19 million RNAseq reads were obtained (Additional file 1).

### Global gene expression pattern in wild type and *ram1*

The petunia genome is predicted to contain at least 32′928 genes [40], of which 22′954 were found to be expressed in the roots (Additional file 2), based on the criterion that they were detected by at least 10 reads in at least one of the four conditions. Based on Baggerly’s test [41], 7′050 genes exhibited a significant difference in gene expression between the mycorrhizal wild type roots and non-mycorrhizal controls (Additional file 2). Among these, 1321 genes were 2-fold induced and 797 were 2-fold repressed. Applying a 5-fold threshold for the expression ratio, 650 genes were induced and 91 genes were repressed, respectively.

In *ram1* mutants, 3992 genes were significantly regulated based on Baggerly’s test, of which 475 were 2-fold induced and 717 2-fold repressed in mycorrhizal roots vs. controls (Additional file 2). Applying a 5-fold threshold, only 201 genes were induced and 51 repressed, respectively (Additional file 2). Of the 5-fold induced genes in wild type and *ram1*, 430 and 151, respectively, exhibited background levels of expression in controls roots, indicating that they are AM-specific. In order to focus on robustly induced AM-dependent genes, the further analysis was restricted to genes with a change in expression ratio of at least 5-fold. The complete set of gene expression data is available in Additional file 3, and the complete list of the 650 genes with the induction ratio >5 is provided in Additional file 4.

### *ram1* shows residual expression of AM-related genes

In order to address commonalities between the expression patterns in wild type and *ram1*, we tested how many genes were induced in both genotypes (Fig. 1c-e). Among the 650 genes induced in the wild type, 182 were also induced in *ram1* (Fig. 1c). Among the 201 genes induced in *ram1*, only 19 were not induced in the wild type. These results suggest that the genes induced in *ram1* represent essentially a subgroup of the genes induced in the wild type. In agreement with this assumption, no gene showed an inverse gene induction ratio (induced in wild type and repressed in *ram1*, or vice versa) (Fig. 1c). No gene was repressed both in wild type and *ram1* (Fig. 1c), consistent with previous findings that gene repression is not a consistent feature in AM-related reprogramming of gene expression [18]. Not only more genes were induced in wild type compared to *ram1*, but also considerably more were specifically expressed in mycorrhizal roots (straight line of points in Fig. 1 d, e; Additional file 2).

### Functional categories induced in mycorrhizal roots

**Fig. 2:**
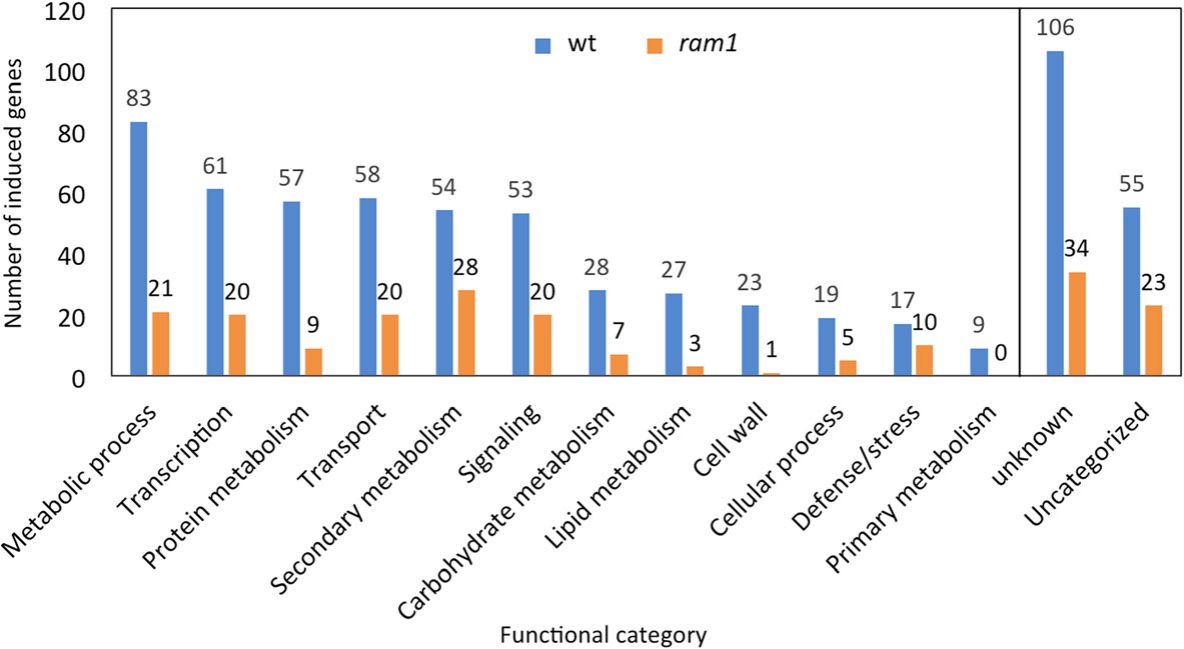
Predicted functional categories of AM-induced genes. Genes induced >5-fold in mycorrhizal wild type roots (*blue*), and *ram1* (*orange*) where sorted according to functional categories based on their Interpro domains and/or function of their closest homologs. Numbers on top of the bars indicate the number of induced genes per category in wild type and *ram1*, respectively

Among the 650 genes induced >5-fold in the wild type, the two highest GO functional categories were ‘metabolic process’ and ‘transcription’, followed by ‘protein metabolic process’, ‘transport’, ‘secondary metabolism’, and ‘signaling’ (Fig. 2). This pattern of induction is compatible with previous transcriptomic studies in various AM hosts [18–24]. Based on the large number of AM-induced genes (>600), it is not surprising that many AM-induced genes belong to the category ‘transcription’. Considering the residual gene induction in *ram1*, a variable fraction of genes were still induced in the mutant to a certain degree (Fig. 2), although in the categories “metabolic process”, “protein metabolism”, lipid metabolism’, and ‘cell wall’, very low numbers of genes were induced in *ram1* (Fig. 2).

In order to appreciate the global changes in gene expression in *ram1* in a more quantitative way, the induction of all AM-induced genes (>5×) was plotted for the wild type and *ram1* ordered by categories as in Fig. 2 (Additional file 5). While induction ratios from 5-fold to over 6000-fold are identically counted as “induced” in Figs. 1 and 2, this representation allows better to compare the effect of *ram1* mutation on AM-related gene induction on a global scale and in a quantitative way. Additional file 5 reveals the strong general reduction in gene expression of most AM-inducible genes in *ram1*. All categories, and most individual genes, exhibited strongly reduced expression levels in *ram1* compared to the wild type, indicating that the majority of all AM-inducible genes depends, directly or indirectly, on RAM1 for induction.

### GO term enrichment analysis among differentially expressed genes in wild type and *ram1*

In order to test which pathways are differentially regulated by AM in the wild type and *ram1*, we determined the fraction of differentially expressed genes in groups that belong to defined GO categories, relative to the total number of genes in the respective categories based on the complete genome sequence. This analysis of GO term enrichment revealed several pathways to be significantly overrepresented in the AM-induced genes (Additional file 6). Based on the statistical significance of over-expressed genes in mycorrhizal pathways, it became apparent that the main metabolic pathways in the two genotypes strongly differ (Additional file 6). Only few pathways that were overrepresented in the wild type were also overrepresented in the mutant. In particular, the highly induced ammonium uptake pathway was not induced in *ram1*, whereas GO categories in protein ubiquitination and in defense and response to biotic stress remained induced in the mutants like in the wild type (Additional file 6). Similarly, the repression in the wild type of sulfate and phosphate transporter pathways, which may reflect the inhibition of the constitutive uptake pathways in mycorrhizal roots, was not observed in *ram1* (Additional file 6). These results indicate that many symbiotic functions are particularly dependent on RAM1, either directly or, indirectly, due to the abnormal development of arbuscules. Thus, these results shed new light on major arbuscule-related cellular pathways, and on their dependence on RAM1.

### Regulation of genes encoding transcription factors

**Fig. 3:**
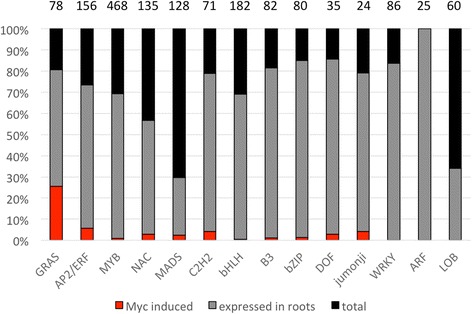
Gene expression of the main transcription factor families. Genes where identified with their Interpro domains. Genes with a mean of more than 10 reads in mycorrhizal and/or control samples where considered as expressed in the roots and are shown in *grey*. AM-induced genes (at least 5-fold induction) are shown in red. The remaining black portion of the bars represents the gene members that are not expressed in roots. Numbers on top of the bars indicate the total number of genes per category

Since transcriptional activation is a central feature of AM, and since the gene category with the second most AM-induced genes was “transcription”, we explored the entire set of genes encoding transcription factors (TFs) in the petunia genome [40], and we determined the fraction of the individual TF classes that are induced in mycorrhizal roots. Most of the AM-induced TFs belonged to the GRAS family of TFs (Additional file 7), where 26% of all members were AM-inducible (Fig. 3). After the GRAS TFs, the class with the second-largest fraction of AM-inducible members was the AP2/ERF-type TFs (Fig. 3; Additional file 7), consistent with previous reports [40]. Among a total of 53 AM-induced TFs, 19 belong to the GRAS family (Additional file 7), 10 are classified as AP/ERF-type TFs, while the remaining 16 TFs are distributed over seven other classes of TFs (Fig. 3; Additional file 7). While the largest TF class in petunia is represented by MYB TFs (Additional file 7), only few of them were induced in AM (Fig. 3; Additional file 7).

### GRAS transcription factors induced in AM

**Fig. 4:**
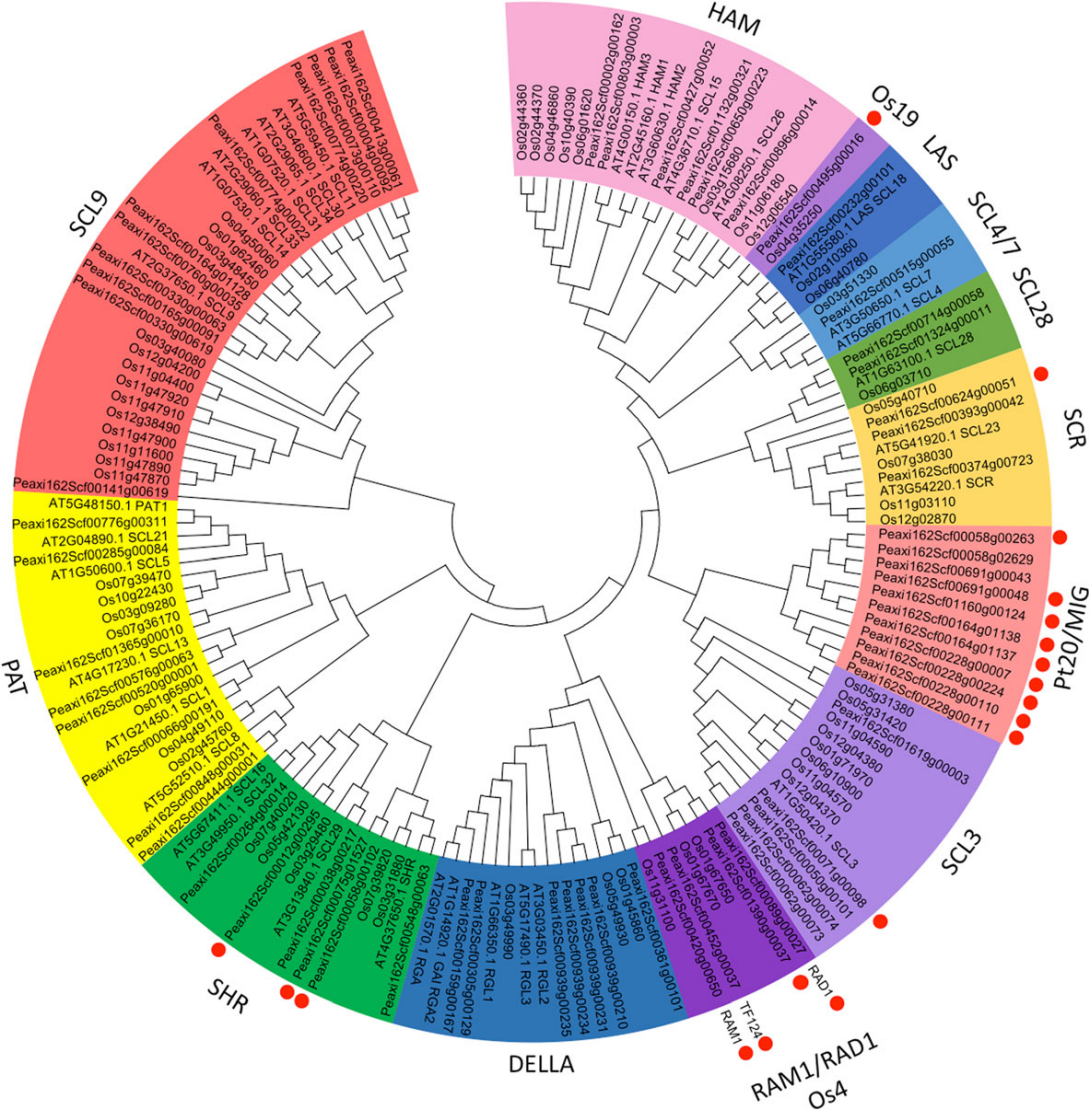
Phylogenetic analysis of the GRAS family. Protein sequences of GRAS proteins from *A. thaliana*, *O. sativa* and *P. axillaris* where processed with MEGA. AM-induced petunia genes analyzed in this study are marked with *red dots*. The phylogeny of GRAS subfamilies with AM-induced members in petunia are shown in more detail in Additional files 7, 8, 9, 10, 11 and 12

GRAS proteins represent a large family of TFs with a wide range of functions in development and plant-microbe interactions [42]. In order to classify the GRAS members regulated during AM symbiosis, the phylogeny of all petunia GRAS-type TFs was established according to [43]. Petunia GRAS proteins can be classified in 13 subfamilies (Fig. 4) [43]. The 19 AM-inducible GRAS proteins are distributed over 6 subfamilies (Fig. 4).

An outstanding group is the Pt20/MIG subfamily, in which nine of the 11 members turned out to be induced in mycorrhizal roots (Fig. 4, Additional file 8). The MIG family was recently found in *M. truncatula* as a group of AM-induced genes with at least one member (MIG1) being required for arbuscule development [38]. The 11 MIG genes of petunia are distributed over 5 contigs in the genome (indicated by similar contig names in Fig. 4), i.e. some of them are arranged in close proximity in the genome, similar to the *M. truncatula* genes MIG1-MIG3 that are organized in a cluster as tandem repeats [38]. The MIG subfamily is specific to dicotyledonous plants species, but is absent from *Arabidopsis* (Additional file 8).

The RAM1/RAD1 subfamily is also absent from *Arabidopsis* but it exists both in monocot and dicot species that are competent to engage in AM (Additional file 9). This phylogenetic pattern is indicative of evolutionary selection for an AM-related function [44–46]. RAM1 and RAD1 belong to two closely related sister clades that each has at least one AM-induced member in *P. hybrida*, *M. truncatula*, and *L. japonicus* (Fig. 4; Additional file 9). A third clade with an AM-related phylogeny is the Os19 subfamily that has members in monocot and dicot species, but not *Arabidopsis* (Additional file 10). Additional AM-induced members where identified in the SCARECORW (SCR) clade (Additional file 11), in the SHORT ROOT (SHR) clade (Additional file 12), as well as in the SCL3 clade (Additional file 13).

Since the GRAS genes were most prominent among the AM-inducible TF genes (Fig. 3), we further explored the expression of a subset of 11 GRAS genes. Quantitative real-time PCR (qPCR) analysis revealed that 8 of them were highly induced, or exclusively expressed, in mycorrhizal roots (Additional file 14). Interestingly, the three remaining genes were significantly repressed in mycorrhizal roots, relative to the controls (Additional file 14). To test whether these GRAS genes may also have a role in aerial organs, we also determined their expression in leaves, buds (shoot tips), stems, petals stamens and carpels. These experiments showed that the AM-induced GRAS genes (RAM1, GRAS1-GRAS6) were primarily or exclusively expressed in mycorrhizal roots (Additional file 15). The AM-repressed GRAS genes (GRAS11-GRAS13), on the other hand, exhibited an ubiquitous expression patterns, including the floral organs. These results show that several GRAS genes are dedicated to AM symbiosis, as it was shown in *L. japonicus* [47].

### Role of RAM1 during early stages of AM

One of the goals of this study was to identify the AM-inducible genes that depend on activation of RAM1, and from this information to interprete the defect of *ram1* mutants in AM symbiosis. In the first report on RAM1, its function has been proposed to be related to early presymbiotic signaling through the action on its target RAM2 [34, 48]. Recent studies, however, suggested that RAM1 might act later by controlling fungal morphogenesis during the formation of arbuscules in cells of the root cortex [35–37]. Since the biological functions of TFs are mediated by their target genes, we assessed well-defined groups of AM-related genes that can serve as markers for distinct stages of the interaction [3].

**Fig. 5:**
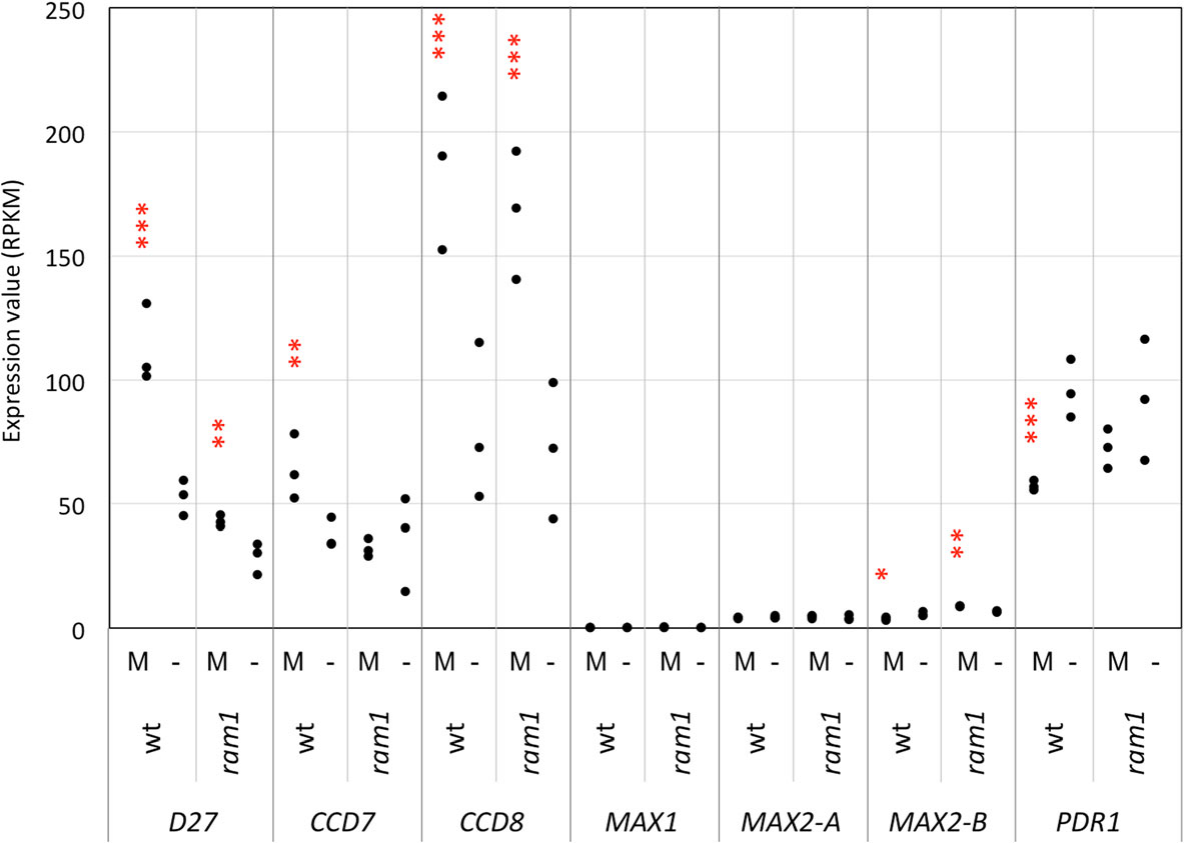
Expression of strigolactone-related genes. Gene expression levels are expressed as RPKM (Reads Per Kilobase per Million mapped reads). The following genes are represented: D27 (Peaxi162Scf00165g00212.1), CCD7 (Peaxi162Scf00377g00829.1), CCD8 (Peaxi162Scf00227g00714.1), MAX1 (Peaxi162Scf00327g00049.1), MAX2-A (Peaxi162Scf00384g00211.1) and MAX2-B (Peaxi162Scf00469g00034.1). Asterisks show significant changes between expression in mycorrhizal roots (M) compared to non-mycorrhizal controls (−) for *p* < 0.05 (*), *p* < 0.01 (**), and *p* < 0.001 (***)

**Fig. 6:**
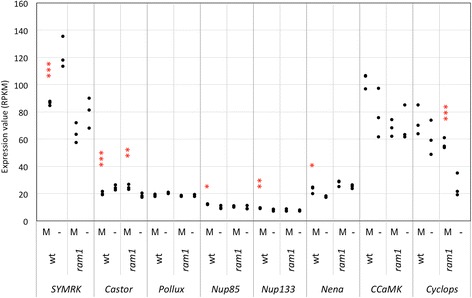
Expression of CSSP genes. Gene expression levels are expressed as RPKM (Reads Per Kilobase per Million mapped reads). The following genes are represented: SYMRK (Peaxi162Scf00703g00117.1), Castor (Peaxi162Scf00883g00321.1), Pollux (Peaxi162Scf00002g02318.1), Nup85 (Peaxi162Scf00227g00812.1), Nup133 (Peaxi162Scf00776g00233.1), Nena (Peaxi162Scf00074g01141.1), CCaMK (Peaxi162Scf00647g00529.1) and Cyclops (Peaxi162Scf02295g00022.1). Asterisks show significant changes between expression in mycorrhizal roots (M) compared to non-mycorrhizal controls (−) for *p* < 0.05 (*), *p* < 0.01 (**), and *p* < 0.001 (***)

The earliest genes with a role in AM are involved in the biosynthesis and in the release of the phytohormone strigolactone (SL), which stimulates AM fungi [10, 11, 49]. A second set of genes is required for signaling upon mutual recognition between the two partners in AM, as well as in root nodule symbiosis, and therefore they are known as CSSP genes [3–5]. Both these groups of genes were only marginally affected at the transcriptional level in *ram1* mutants, indicating that their expression does not depend on RAM1 (Figs. 5 and 6).

### Role of RAM1 during arbuscule development

Many of the AM-induced genes are thought to have a specific symbiosis-related function. Some of them appear to function in cellular infection (penetration) and in intracellular accommodation of AM fungi [3]. Among these, VAPYRIN and EXO70i are the best characterized examples [50–53].

At later stages, the symbiotic function of arbuscules requires the activity of specific nutrient transporters such as the phosphate transporter PT4, which is localized to the periarbuscular membrane, where it mediates the transfer of inorganic phosphate delivered by the fungus into the symbiotic interface [27]. Other proteins, such as STR and STR2 have been shown to be expressed in cells with arbuscules and to be localized to the periarbuscular membrane as well, but their function in symbiosis is unknown [54]. Finally, for genes such as *RAM2*, it is clear that they are expressed in cells with arbuscules, while the function and the localization of the protein is elusive. Nevertheless, all these genes are established markers for cells with functional arbuscules, and they are essential for AM.

**Fig. 7:**
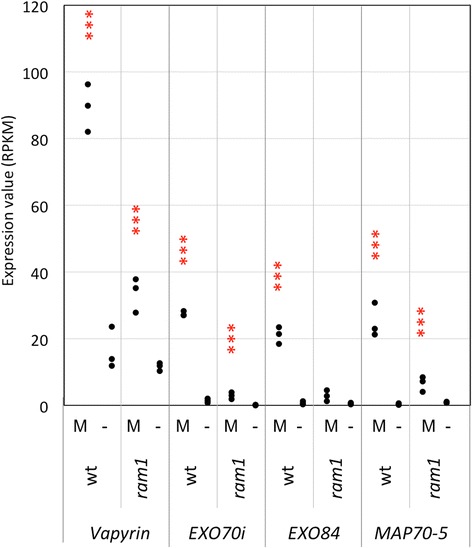
Expression of genes related to cellular functions in AM. Gene expression levels are expressed as RPKM (Reads Per Kilobase per Million mapped reads). The following genes are represented: Vapyrin (Peaxi162Scf00575g00001.1), Petunia EXO70i orthologue (Peaxi162Scf00081g02225.1), the exocyst complex component EXO84 (Peaxi162Scf00415g00042.1) and the microtubule-associated protein 70-5 (MAP70-5, Peaxi162Scf00008g00212.1). Asterisks show significant changes between expression in mycorrhizal roots (M) compared to non-mycorrhizal controls (−) for *p* < 0.05 (*), *p* < 0.01 (**), and *p* < 0.001 (***)

In order to test for the function of RAM1 at later stages of AM, we assessed the expression of later marker genes in *ram1* mutants. The markers VAPYRIN and EXO70i, which are involved in arbuscule morphogenesis and branching, show a basal level of expression and are induced in mycorrhizal roots [50, 52, 53]. In *ram1*, they showed a similar behavior as in the wild type, but with considerably lower levels of induction (Fig. 7). A second component of the exocyst, EXO84, as well as an AM-responsive gene encoding a microtubule-associated protein (MAP gene), which might be involved in arbuscule morphogenesis [55, 56], showed very similar expression patterns as EXO70i (Fig. 7).

**Fig. 8:**
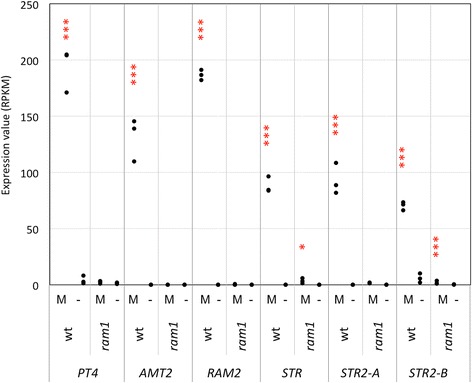
Expression of arbuscule-related genes. Gene expression levels are expressed as RPKM (Reads Per Kilobase per Million mapped reads). The following genes are represented: PT4 (Peaxi162Scf00618g00532.1), ammonium transporter AMT2 (Peaxi162Scf00341g00514.1), RAM2 orthologue (Peaxi162Scf00129g00625.1), STR (Peaxi162Scf00282g00221.1) and two STR2 (Peaxi162Scf00286g00240.1, Peaxi162Scf00378g00624.1). Asterisks show significant changes between expression in mycorrhizal roots (M) compared to non-mycorrhizal controls (−) for *p* < 0.05 (*), *p* < 0.01 (**), and *p* < 0.001 (***)

In contrast to this expression pattern, the expression of marker genes with late functions in arbusculated cells is essentially restricted to cells with functional arbuscules. In particular, PT4, AMT2, RAM2, STR, and the duplicated STR2A and STR2B, which are not expressed in non-mycorrhizal control roots (Fig. 8), were not expressed in mycorrhizal *ram1* mutant roots (Fig. 8). Hence, the induction of these late AM-related marker genes depends on *RAM1* and/or fully developed arbuscules. Taken together, these results indicate that the induction of late AM-specific genes strictly depends on *RAM1*, whereas earlier genes, including genes required for signaling, cellular penetration, and intracellular accommodation show little or intermediate dependence on *RAM1*. Based on the essential function during AM of RAM1-dependent genes such as *PT4*, *RAM2*, *STR* and *STR2*, the defect of *ram1* mutants in arbuscule formation and in the establishment of a functional symbiosis can be explained by the lack of induction of these genes in *ram1*.

In order to obtain indepent evidence for the gene expression patterns described above, we employed qPCR. Selected representatives were chosen from the four gene groups based on their potential importance for AM symbiosis. D27 and CCD8 are involved in SL biosynthesis, SYMRK and CYCLOPS are components of the CSSP, VAPYRIN is essential for infection, whereas PT4 and RAM2 are essential for arbuscule functioning. Comparison of their expression showed that the early genes (D27, CCD8, SYMRK, CYCLOPS) were only marginally affected by the *ram1* mutation, whereas PT4 and RAM2 induction in mycorrhizal roots was undetectable in *ram1* mutants, as in non-colonized roots. These results are in line with our RNAseq data (Figs. 5, 6, 7 and 8), as well as with the literature on RAM1 [34–37, 47].

## Discussion

### Establishment of AM

Endosymbioses of plants such as AM and RNS involve intracellular accommodation of fungal and bacterial mutualists, respectively, in root cells. In the case of RNS, a new organ, the nodule, is formed which is optimized for efficient symbiotic function. In order to allow root cells to be infected and to establish the cellular machinery required for new symbiosis-related functions, such as suppression of defense, intracellular accommodation of the microbe, and nutrient exchange, the host cells need to be fundamentally reprogrammed. The reprogramming of root cells during the establishment of AM has been described in several host species at the cellular level [55, 57–60], as well as at the transcriptomic level [18–24]. Both approaches revealed considerable commonalities of AM in different hosts, indicating a high degree of conservation. Consistent with these observations, a conserved signalling pathway, the CSSP, is required for the development of AM as well as of RNS [3–5].

### Transcriptional networks in AM

The conserved patterns of gene induction in AM [18] suggests that a conserved machinery reprograms root cells for symbiosis, and indeed, the conserved function of the GRAS protein RAM1 in gene regulation and arbuscule morphogenesis in various AM-competent angiosperms indicates that RAM1 may represent a central component in this machinery [34–37, 47]. Little is known about the regulatory cascade that induces AM-related genes downstream of the CSSP. The best-characterized TFs with a role in AM symbiosis are RAM1 and CYCLOPS (CYC). Recent evidence suggests that CYC is activated by direct interaction with CCaMK, which responds to calcium spiking in the nucleus [17]. Activated CYC targets the promoter motif GCCGGC that occurs in many AM-related genes of *M. truncatula* [45]. In particular, CYC induces the transcription of RAM1, which in turn can activate downstream AM-related target genes [34–37, 47].

### Role of GRAS transcription factors in AM symbiosis

Transcriptional reprogramming often relies on cascades of TFs with upstream master regulators, and downstream transcriptional networks involving multiple TFs for functionally relevant groups of target genes. CYC is likely to function as a master switch, since it is directly activated by the CSSP, and it is itself not transcriptionally induced during AM, just as the other components of the CSSP (Fig. 6). Hence, it does not require transcriptional activation for its function in AM. In contrast, *RAM1* is strongly induced during AM. It is a direct target of CYC [36], and the fact that *cyc* mutants transformed with a *RAM1* overexpression construct exhibit restored AM colonization [36] suggests that CYC acts primarily through *RAM1*. Therefore, RAM1 may represent a subordinate master regulator that activates the donwstream AM-related genes required for AM functioning. Indeed, previous results have shown that induction of several AM marker genes depends on *RAM1* [34–37, 47].

Here, we show that GRAS proteins represent an important part of the transcriptional network required for AM, as it is the case for *L. japonicus* [47]. An interesting case is the MIG family of TFs, which represent a dicot-specific GRAS subfamily with AM-induction in *M. truncatula*, *L. japonicus*, as well as in petunia (Additional file 8). The fact that *MIG* genes are arranged in tandem repeats both, in *M. truncatula* as well as in petunia, suggests that they have been subject to repeated gene duplications. Interestingly, the phylogenetic patterns between the two species suggests that the MIG duplications were species-specific and therefore more recent then the common ancestor of the two species (Additional file 8). Although DELLA proteins have been shown to be important for AM in monocots and dicots [36, 61, 62], no DELLA gene was induced in petunia (Fig. 4), indicating that their expression may be constitutively active independent of the mycorrhizal status.

### RAM1 regulates late genes in AM with a role in symbiotic functioning

We have used several sets of marker genes for specific stages of AM to map the point of action of RAM1 in AM. Based on our comparative transcriptomic analysis, RAM1 has no role in the regulation of early genes such as the presymbiotic SL-related genes, and the CSSP genes (Figs. 5 and 6). This conclusion is consistent with a role of RAM1 downstream of CYC and downstream of the CSSP. A partial dependence of the infection markers VAPYRIN and EXO70i (Fig. 7) suggests that RAM1 may contribute, directly or indirectly to the induction of these genes.

Considering later genes that encode proteins with essential roles in nutrient exchange and in arbuscule functioning, and that are expressed only in cells with developing arbuscules, it appears that RAM1 directly or indirectly controls a wide array of these target genes (Additional file 3; Fig. 8). Interestingly, recent evidence suggests that some of these genes (in particular RAM2, STR, and STR2) may be related to the supply of the fungal partner with lipid-related substrates [63, 64]. Taken together, these findings and results from previous studies [34–37, 47], suggest that RAM1 acts as a central element in the transcriptional activation of arbuscule-related genes.

## Conclusion

*RAM1* encodes a GRAS transcription factor that is essential for the induction of the majority of AM-inducible genes. In particular, late arbuscule-related genes that encode essential functions in nutrient exchange are strictly dependent on RAM1. Since these genes are required for the establishment of AM, the defect of *ram1* mutants in AM development can be explained by the lack of induction of these essential target genes. An interesting outcome of our study was the observation that many TFs, in particular GRAS TFs are induced in mycorrhizal roots, of which the majority belongs to phylogenetic clades that are absent from the non-mycorrhizal model plant *Arabidopsis*, thus indicating that they may have been under selection for a function in symbiosis.

## Methods

### Plant material, growth conditions, and inoculation

*Petunia hybrida* wild type W115 and petunia *ram1-2* [37] seeds were surface-sterilized with sodium hypochlorite (1.3%, 10 min), rinsed five times in sterile water and germinated on seedling substrate (Klasmann, http://www.klasmann-deilmann.com). After 4 weeks, plantlets were transferred to 10x10cm pots containing a sterilized mixture of 75% sand with 25% unfertilized soil and inoculated with around 30 g of soil inoculum of *Rhizophagus irregularis* (MUCL 43204). Plants were grown in growth chambers with a day/night cycle of 12 h (25 °C)/12 h (20 °C). Plants were fertilized weekly with a solution containing 3 mM MgSO_4_, 0.75 mM KNO_3_, 0.87 mM KCl, 0.2 mM KH_2_PO_4_, 1.52 mM Ca(NO_3_)_2_, 0.02 mM NaFeEDTA, 11 μM MnSO_4_, 1 μM ZnSO_4_, 30 μM H_3_BO_3_, 0.96 μM CuSO_4_, 0.03 μM (NH_4_)_6_Mo_7_O_24_, and 0.01 μM Na_2_MoO_4_.

### Sample preparation and analysis

Plant roots were harvested 15 days post infection (dpi). Roots were removed from substrate under tap water and cut into ~1 cm small pieces. Two subsamples of about 100 mg were immediately frozen in liquid nitrogen, stored at −80 °C and lyophilised. One subsample of about 100 mg was taken for root colonization measurements.

Total RNA was extracted from lyophilized roots using the guanidinium thiocyanate-phenol-chloroform method [65] and DNAse treated on column (DNA, RNA and protein purification, Macherey-Nagel, Switzerland). Total RNA was quantified with the Qbit RNA BR Assay kit and purity was estimated using the Nanodrop (ND-1000, Witec, Switzerland). Preparation of 12 libraries and 2 × 100 bp pair-end Illumina HiSeq mRNA sequencing (RNA-Seq) was performed by Beckman Coulter Genomics (Danvers, MA, USA). RNAseq raw data were submitted to GEO (https://www.ncbi.nlm.nih.gov/geo/query/acc.cgi?token=gzofymqunhctjcd&acc=GSE96896).

### Sequence processing

Raw reads were trimmed for quality and aligned to the *Petunia axillaris* v4 reference transcripts from solgenomics (https://solgenomics.net) using the suite CLC Genomics Workbench v9. For mapping, the minimum length fraction was 0.9, the minimum similarity fraction 0.9 and the maximum number of hits for a read was set to 10. The unique and total mapped number of reads for each transcript was determined, and then normalized to RPKM (Reads Per Kilobase of exon model per Million mapped reads). Intact pairs were counted as two, broken pairs as one. Fold change values were calculated by proportion-based test statistics [41] with a False Discovery Rate (FDR) correction for multiple testing [66]. Genes were considered differentially expressed if the induction ratio was either 2-fold induced or repressed (−2 ≤ or ≥2) with an FDR-corrected *p*-value of *p* < 0.05, and if the difference between mycorrhizal and control tretments was at least 10 reads.

Gene set enrichment was performed with CLC Genomic Workbench by using the unconditional GOstats test of [67] based on the *Petunia axillaris* v4 annotation. This test measures the extent to which the annotation categories of features in up (≥2) or down (≤ − 2) differentially expressed gene lists are over- or under-represented relative to those of the features in the total Petunia gene repertoire, The “*p*-value” corresponds to the tail probability of the hyper geometric distribution. This value indicates whether functionally related gene groups defined by GO terms are significantly over- or underrepresented among genes that are differentially regulated compared to the gene set in the complete genome.

### Real time PCR

Reverse transcription was performed on 1 μg of RNA with the Omniscript reverse transcription (RT) kit (Qiagen) using a mix of oligo(dT) and random primers (Promega) according to the manufacturer’s instructions. Quantitative Real-Time PCR was performed on a mic qPCR cycler (Bio Molecular Systems) with the SensiMix SYBR Hi-ROX Kit (Bioline). Relative expression was calculated using the GAPDH gene as reference gene [39]. Results shown in Additional files 14 and 15 were obtained with RNA isolated from shoot tips (bud), leaves, pistils, stems, stamens, petals, control roots (roots-NM), and mycorrhizal roots harvested 5 weeks after inoculation with *R. irregularis* MUCL43204 (roots-M). Results shown in Additional file 16 were obtained using the same RNA samples as the RNAseq experiment.

### Phylogenetic analysis

For phylogenetic analysis, the GRAS amino acid sequences were aligned with ClustalW (http://www.ebi.ac.uk/Tools/msa/clustalw2/) using the following multiple alignment parameters: gap opening penalty 15, gap extension penalty 0.3, and delay divergent sequences set to 25%; and the Gonnet series was selected as the protein weight matrix. Neighbor joining trees were constructed using Poisson correction model for distance computation in MEGA4 [68]. Bootstrap analysis was carried out with 1000 replicates. Branch lengths (drawn in the horizontal dimension only) are proportional to phylogenetic distances.

## Additional files

Additional file 1: Summary table of RNAseq experiment. (PDF 55 kb)

Additional file 2: Summary table of gene expression changes in wild type and *ram1.* Induction and repression ratios are expressed as the ratio between mycorrhizal roots and their respective non-mycorrhizal controls. (PDF 40 kb)

Additional file 3: Table listing the complete gene data set of this study. (XLSX 4836 kb)

Additional file 4: Table listing the genes induced at least 5-fold in the wild type and their expession in *ram1*. All AM-incucible genes were sorted according to their predicted functional category as in Fig. 2. Expression values (RPKM) are given for mycorrhizal wild type (wt-M_RPKM means), wild type control roots (wt-NM_RPKM means), mycorrhizal *ram1* (ram1-M_RPKM means), and *ram1* control roots (ram1-M_RPKM means). The respective induction ratio (AM vs. control roots) are given for the wildtype (column P) and *ram1* (Column BB). (XLSX 542 kb)

Additional file 5: Figure showing the global comparison of AM-dependent gene expression in wild type and *ram1*. Gene induction ratios for AM-induced genes were plotted for wild type (grey), and *ram1* (black) for the AM-inducible genes listed in Additional file 4. An arrow indicates the start of the genes belonging to group 7 (Carbohydrate metabolism), which were only moderately induced (<50-fold), relative to the other groups. (PDF 134 kb)

Additional file 6: Table showing the global comparison of GO terms induced or repressed in wild type and *ram1*. Genes were assigned to biochemical and cellular functions based on common GO terms. Only GO terms with significant overrepresentation (*p* < 0.05) among induced or repressed genes are shown. (XLSX 17 kb)

Additional file 7: Table of AM-inducible transcription factors. AM-induced transcription factor genes were classified according to their sequence features. The corresponding induction pattern in *Lotus japonicus* is given for comparison [47]. (PDF 50 kb)

Additional file 8: Figure of phylogenetic tree of GRAS proteins in the AM-specific *Pt20*/*MIG* subfamily. AM-induced genes from *P. axillaris* (Peaxi), *M. truncatula* (Medtr), and *L. japonicus* (Lojap) are marked with red circles; the functionally tested *MIG1* gene from *M. truncatula* is marked with a blue circle. The closest homologue in *A. thaliana* (AT) is highlighted with a red frame. Potri: *Populus trichocarpa*. The distance bar indicates substitutions per site. (PDF 62 kb)

Additional file 9: Figure of phylogenetic tree of GRAS proteins in the AM-specific *RAD1* and *RAM1* subfamilies. AM-induced genes are marked with red circles; functionally tested homologues from *P. axillaris* (Peaxi) *M. truncatula* (Medtr) and *L. japonicus* (Lojap) are marked with blue circles. The closest homologue in *A. thaliana* (AT) is highlighted with a red frame. Potri: *P. trichocarpa*; Sobic: *S bicolor*; Bradi: *Brachipodium distachyon*; Os: *O. sativa*). The distance bar indicates substitutions per site. (PDF 61 kb)

Additional file 10: Figure of phylogenetic tree of GRAS proteins in the AM-specific Os19 subfamily. AM-induced genes in *L. japonicus* (Lojap) and *P. axillaris* (Peaxi) are marked with red circles. The closest homologue in *A. thaliana* (AT) is highlighted with a red frame. Potri: *P. trichocarpa*; Sobic: *S. bicolor*; Bradi: *B. distachyon*; Os: *O. sativa*). The distance bar indicates substitutions per site. (PDF 58 kb)

Additional file 11: Figure of phylogenetic tree of GRAS proteins in the SCARECROW subfamily. AM-induced genes from *P. axillaris*, (Peaxi), *M. truncatula* (Medtr), and *L. japonicus* (Lojap) are marked with red circles. The closest homologue in *A. thaliana* (AT) is highlighted with a red frame. Potri: *P. trichocarpa*; Sobic: *S bicolor*; Bradi: *B. distachyon*; Os: *O. sativa*). The distance bar indicates substitutions per site. (PDF 59 kb)

Additional file 12: Figure of phylogenetic tree of GRAS proteins in the SHORT ROOT subfamily. AM-induced genes from *P. axillaris* (Peaxi), *M. truncatula* (Medtr) and *L. japonicus* (Lojap) are marked with red circles. The functionally characterized NSP1 gene from *M. truncatula* is marked with a blue circle. The closest homologues in *A. thaliana* (AT) are highlighted with red frames. Potri: *P. trichocarpa*; Sobic: *S bicolor*; Bradi: *B. distachyon*; Os: *O. sativa*). The distance bar indicates substitutions per site. (PDF 63 kb)

Additional file 13: Figure of phylogenetic tree of GRAS proteins in the SCARECROW-LIKE3 subfamily. AM-induced genes from *P. axillaris* (Peaxi), *M. truncatula* (Medtr), and *L. japonicus* (Lojap) are marked with red circles. The closest homologue in *A. thaliana* (AT) is highlighted with a red frame. Potri: *P. trichocarpa*; Sobic: *S bicolor*; Bradi: *B. distachyon*; Os: *O. sativa*). The distance bar indicates substitutions per site. (PDF 62 kb)

Additional file 14: Expression of GRAS genes in mycorrhizal roots. Expression analysis by qPCR of various GRAS transcription factor genes in mycorrhizal roots (dark grey columns) and control roots (light grey columns). In all cases, the expression was significantly different. Identities and gene names of GRAS genes can be found in Additional file 3. (PDF 56 kb)

Additional file 15: Expression of GRAS genes in various aerial tissues. Global expression analysis by qPCR of various GRAS transcription factor genes in tissues collected from mycorrhizal roots (Root-M), control roots (Root-NM), shoot tips (buds), and various aerial organs (Leaf, Pistil, Stem, Stamen, Petal). Note logarithmic scale of y-axis. Identities and gene names of GRAS genes can be found in Additional file 3. (PDF 96 kb)

Additional file 16: Expression of AM-related genes involved at different stages of AM interaction. Expression analysis by qPCR of D27, CCD8, SYMRK, VAPYRIN, PT4, and RAM2 in wild type (dark grey columns) and *ram1* mutants (light grey columns) with the AM fungus *R. irregularis* (Ri) or in nonmycorrhizal controls. Note logarithmic scale of y-axis. Identities and gene names of GRAS genes can be found in Additional file 3. (PDF 40 kb)

## Abbreviations

AM: Arbuscular mycorrhiza
CCaMK: Calcium- and calmodulin-dependent protein kinase
CSSP: Common symbiosis signalling pathway
CYC: CYCLOPS
GRAS: GAI, RGA, SCR
RAM1: Required for arbuscular Mycorrhiza1
SL: Strigolactone
STR: Stunted arbuscule
TF: Transcription factor
