# Supplementary material for: Role of the GRAS transcription factor ATA/RAM1 in the transcriptional reprogramming of arbuscular mycorrhiza in Petunia hybrida
Source: BMC Genomics. 2017 Aug 8;18:589. doi: 10.1186/s12864-017-3988-8 (PMC5549340; doi:10.1186/s12864-017-3988-8)
Supplement: Supplementary file 1 — Summary table of RNAseq experiment. (PDF 55 kb) [file 12864_2017_3988_MOESM1_ESM.pdf]

Additional File 1: General information on RNAseq experiment

	wt		*ram1*	
	NM	M	NM	M
Mean number of reads	18 515 472	18 789 580	19 718 736	19 010 779
% fungal reads	N.A	6.360 ± 0.020	N.A	2.557 ± 0.003
% colonisation	N.A	20 ± 5 %	N.A	15 ± 8 %
