# Supplementary material for: Role of the GRAS transcription factor ATA/RAM1 in the transcriptional reprogramming of arbuscular mycorrhiza in Petunia hybrida
Source: BMC Genomics. 2017 Aug 8;18:589. doi: 10.1186/s12864-017-3988-8 (PMC5549340; doi:10.1186/s12864-017-3988-8)
Supplement: Supplementary file 2 — Summary table of gene expression changes in wild type and ram1. Induction and repression ratios are expressed as the ratio between mycorrhizal roots and their respective non-mycorrhizal controls. (PDF 40 kb) [file 12864_2017_3988_MOESM2_ESM.pdf]

Additional File 2: Number of genes per conditions

Total genes	32928	
expressed in roots	22954	
	wt	*ram1*
induced ≥ 2	1321	475
induced ≥ 5	650	201
repressed ≤ 2	797	717
repressed ≤ 5	91	51
significantly regulated	7050	3992
induced ≥ 5 AM-specific	430	151
