# Supplementary figures and images for: Role of the GRAS transcription factor ATA/RAM1 in the transcriptional reprogramming of arbuscular mycorrhiza in Petunia hybrida
Source: BMC Genomics. 2017 Aug 8;18:589. doi: 10.1186/s12864-017-3988-8 (PMC5549340; doi:10.1186/s12864-017-3988-8)

## Additional File 5: Genes induced at least 5-fold in the wild type and their expression in *ram1*

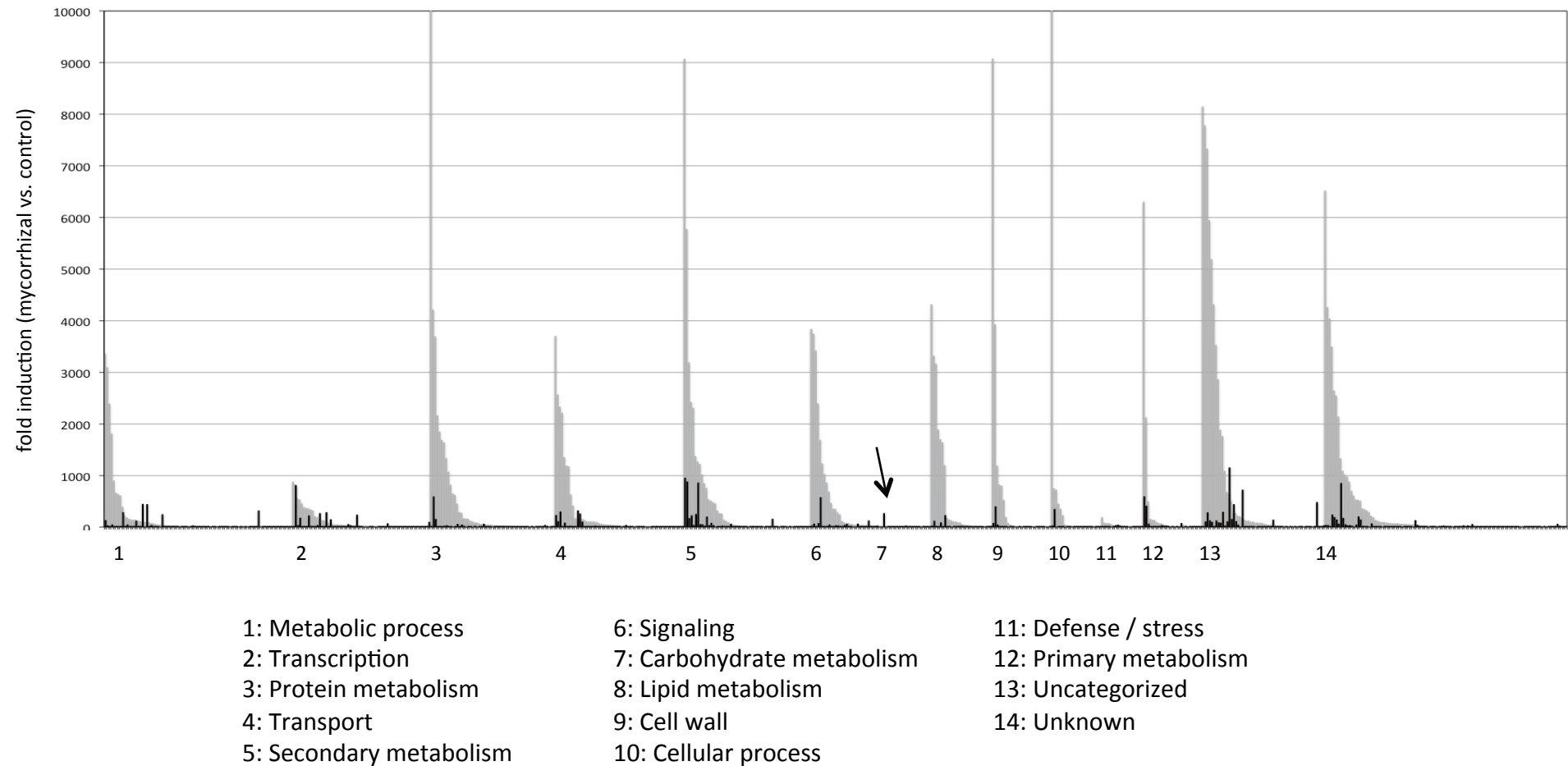

Supplement: Supplementary file 5 — Figure showing the global comparison of AM-dependent gene expression in wild type and ram1. Gene induction ratios for AM-induced genes were plotted for wild type (grey), and ram1 (black) for the AM-inducible genes listed in Additional file 4. An arrow indicates the start of the genes belonging to group 7 (Carbohydrate metabolism), which were only moderately induced (<50-fold), relative to the other groups. (PDF 134 kb) [file 12864_2017_3988_MOESM5_ESM.pdf]

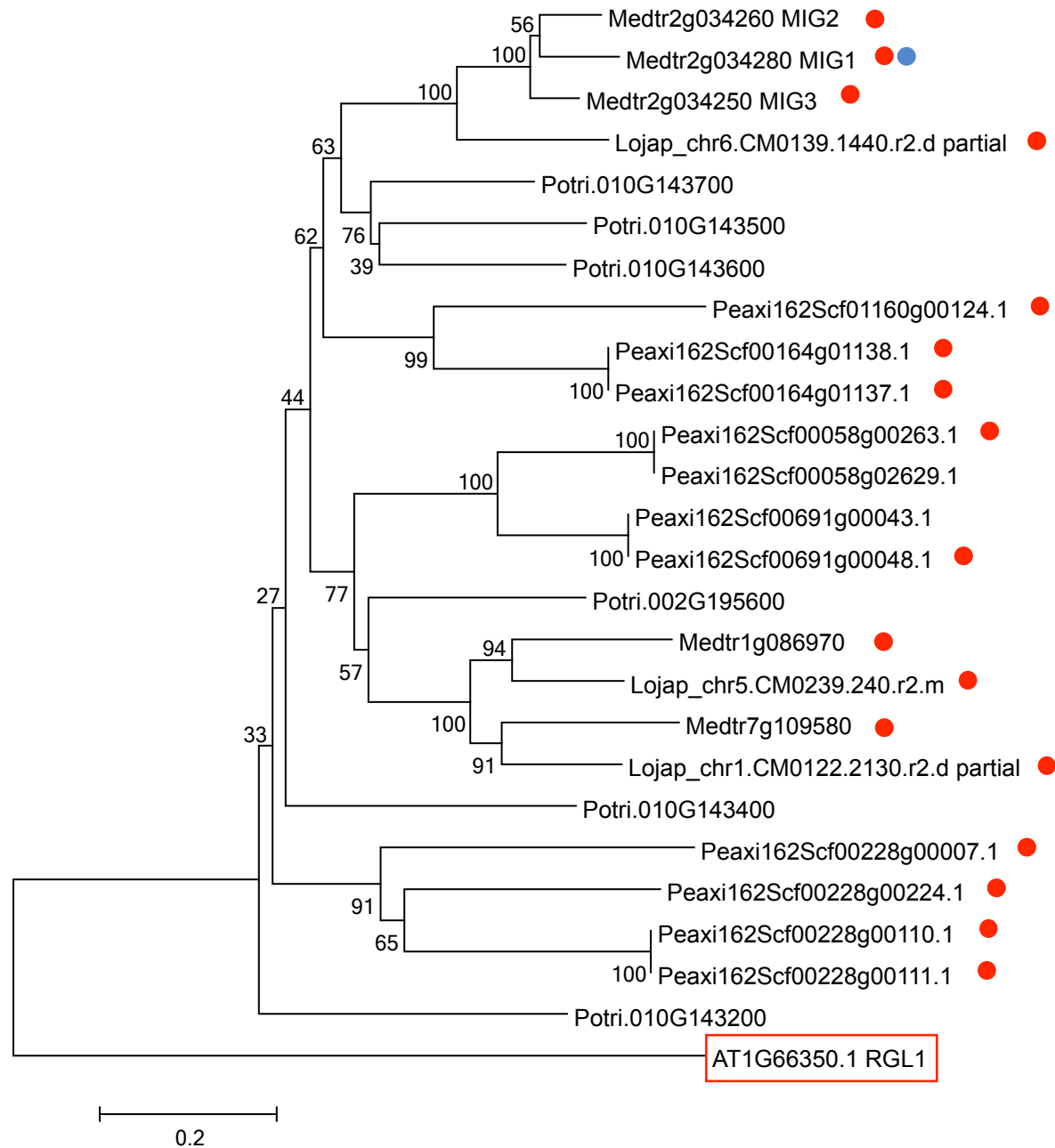

Supplement: Supplementary file 8 — Figure of phylogenetic tree of GRAS proteins in the AM-specific Pt20/MIG subfamily. AM-induced genes from P. axillaris (Peaxi), M. truncatula (Medtr), and L. japonicus (Lojap) are marked with red circles; the functionally tested MIG1 gene from M. truncatula is marked with a blue circle. The closest homologue in A. thaliana (AT) is highlighted with a red frame. Potri: Populus trichocarpa. The distance bar indicates substitutions per site. (PDF 62 kb) [file 12864_2017_3988_MOESM8_ESM.pdf]

## Additional File 9

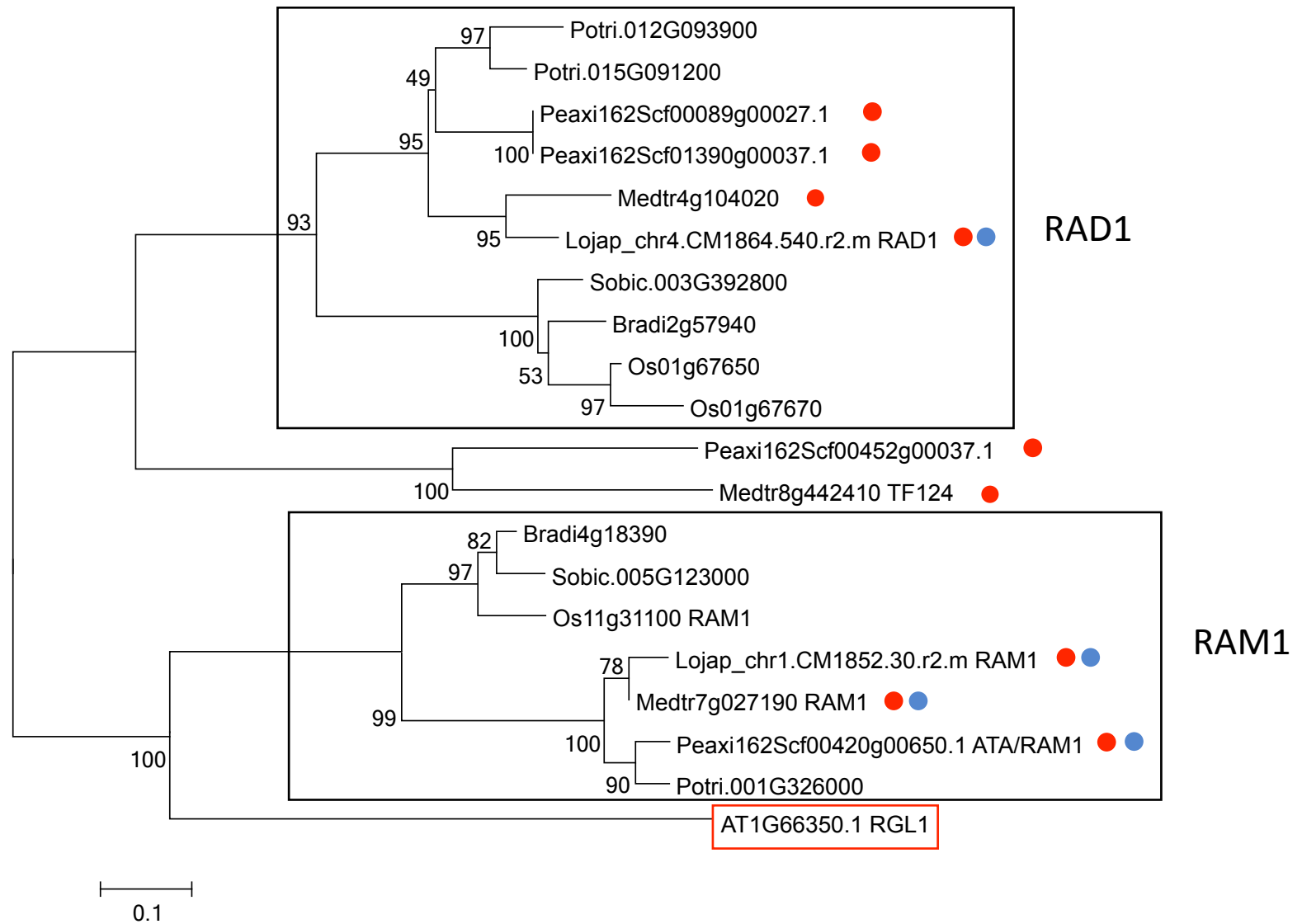

Supplement: Supplementary file 9 — Figure of phylogenetic tree of GRAS proteins in the AM-specific RAD1 and RAM1 subfamilies. AM-induced genes are marked with red circles; functionally tested homologues from P. axillaris (Peaxi) M. truncatula (Medtr) and L. japonicus (Lojap) are marked with blue circles. The closest homologue in A. thaliana (AT) is highlighted with a red frame. Potri: P. trichocarpa; Sobic: S bicolor; Bradi: Brachipodium distachyon; Os: O. sativa). The distance bar indicates substitutions per site. (PDF 61 kb) [file 12864_2017_3988_MOESM9_ESM.pdf]

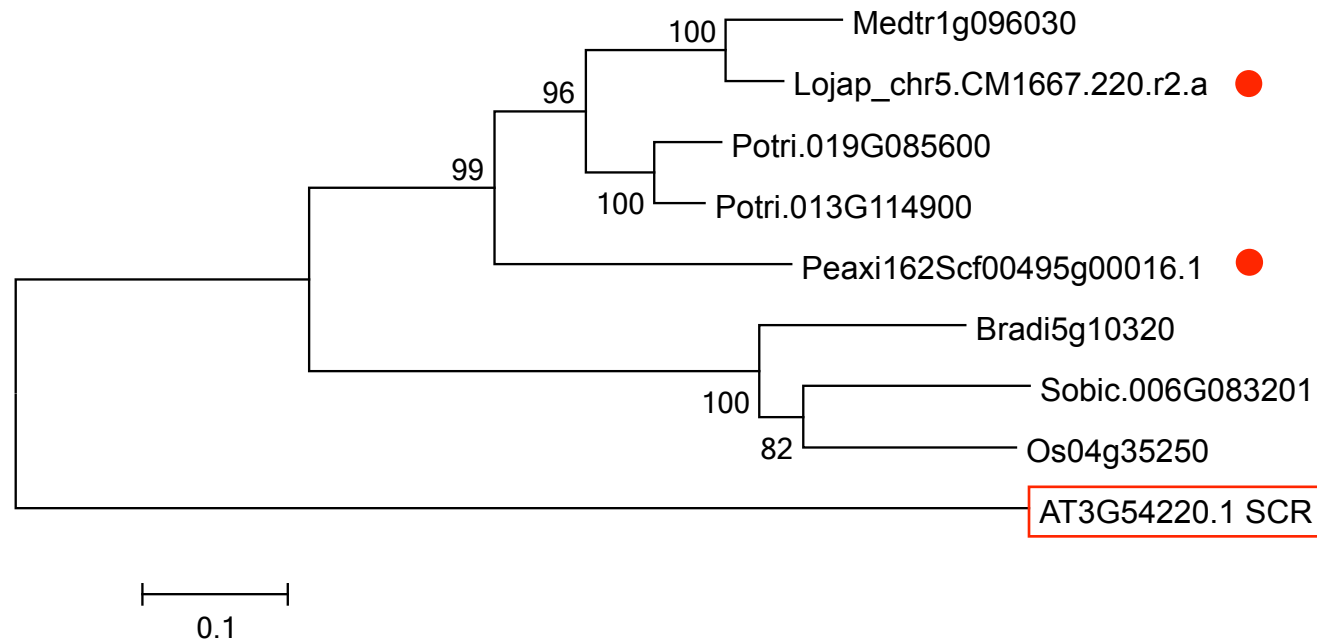

Supplement: Supplementary file 10 — Figure of phylogenetic tree of GRAS proteins in the AM-specific Os19 subfamily. AM-induced genes in L. japonicus (Lojap) and P. axillaris (Peaxi) are marked with red circles. The closest homologue in A. thaliana (AT) is highlighted with a red frame. Potri: P. trichocarpa; Sobic: S. bicolor; Bradi: B. distachyon; Os: O. sativa). The distance bar indicates substitutions per site. (PDF 58 kb) [file 12864_2017_3988_MOESM10_ESM.pdf]

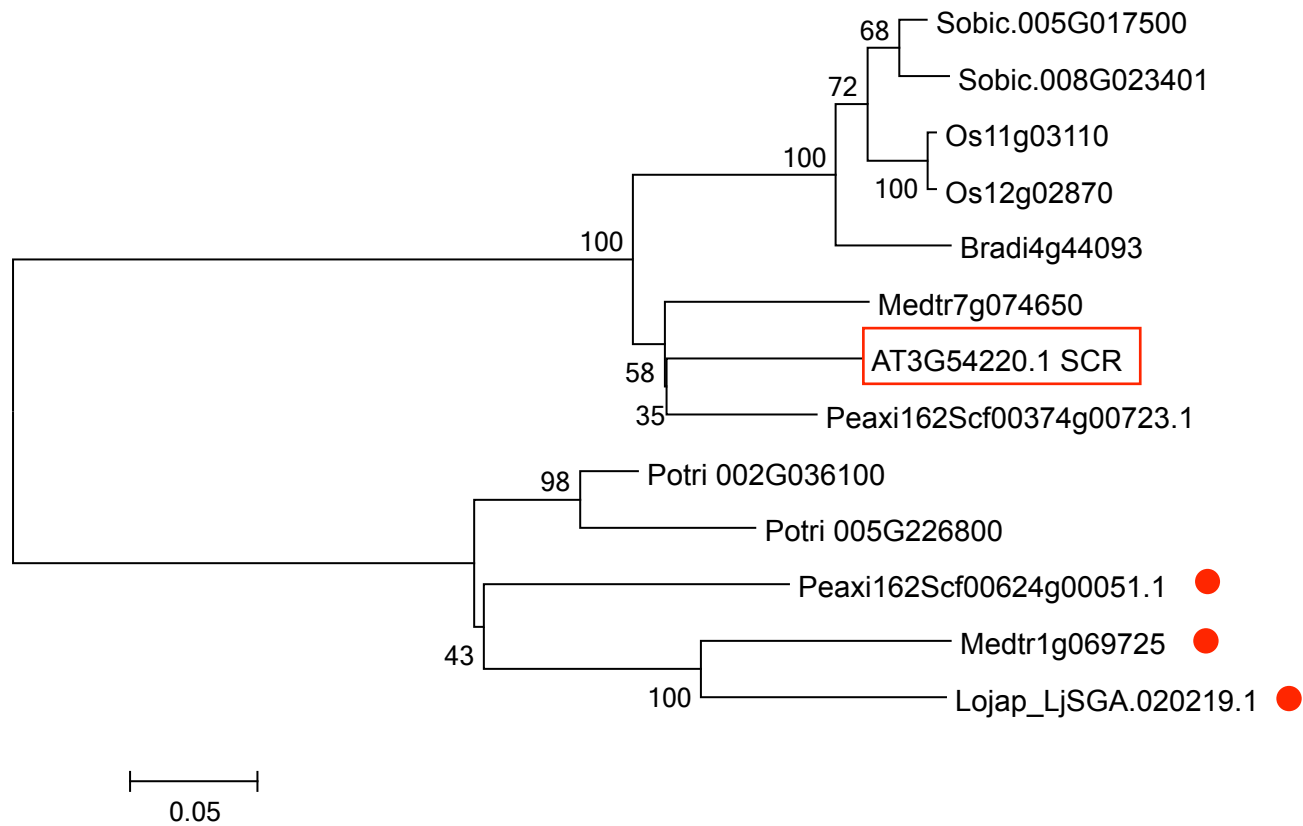

Supplement: Supplementary file 11 — Figure of phylogenetic tree of GRAS proteins in the SCARECROW subfamily. AM-induced genes from P. axillaris, (Peaxi), M. truncatula (Medtr), and L. japonicus (Lojap) are marked with red circles. The closest homologue in A. thaliana (AT) is highlighted with a red frame. Potri: P. trichocarpa; Sobic: S bicolor; Bradi: B. distachyon; Os: O. sativa). The distance bar indicates substitutions per site. (PDF 59 kb) [file 12864_2017_3988_MOESM11_ESM.pdf]

## Additional File 12

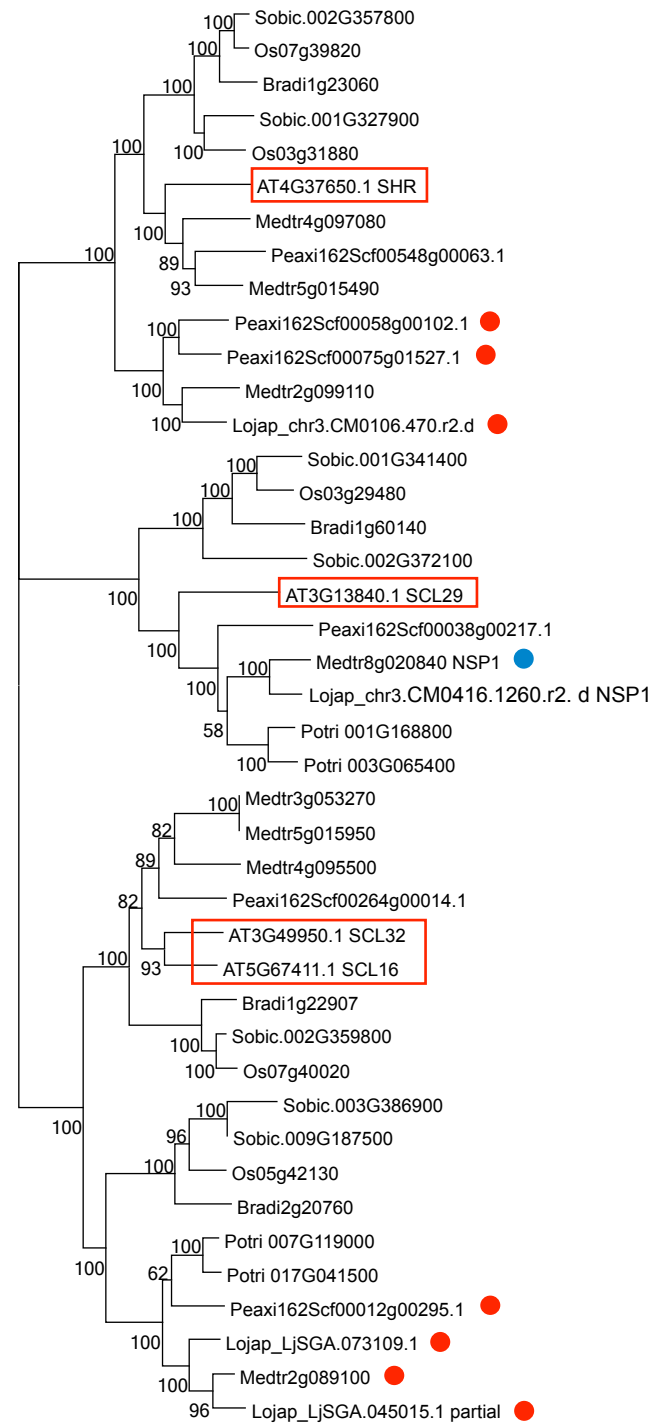

SHR

Supplement: Supplementary file 12 — Figure of phylogenetic tree of GRAS proteins in the SHORT ROOT subfamily. AM-induced genes from P. axillaris (Peaxi), M. truncatula (Medtr) and L. japonicus (Lojap) are marked with red circles. The functionally characterized NSP1 gene from M. truncatula is marked with a blue circle. The closest homologues in A. thaliana (AT) are highlighted with red frames. Potri: P. trichocarpa; Sobic: S bicolor; Bradi: B. distachyon; Os: O. sativa). The distance bar indicates substitutions per site. (PDF 63 kb) [file 12864_2017_3988_MOESM12_ESM.pdf]

Additional File 13

SCL3

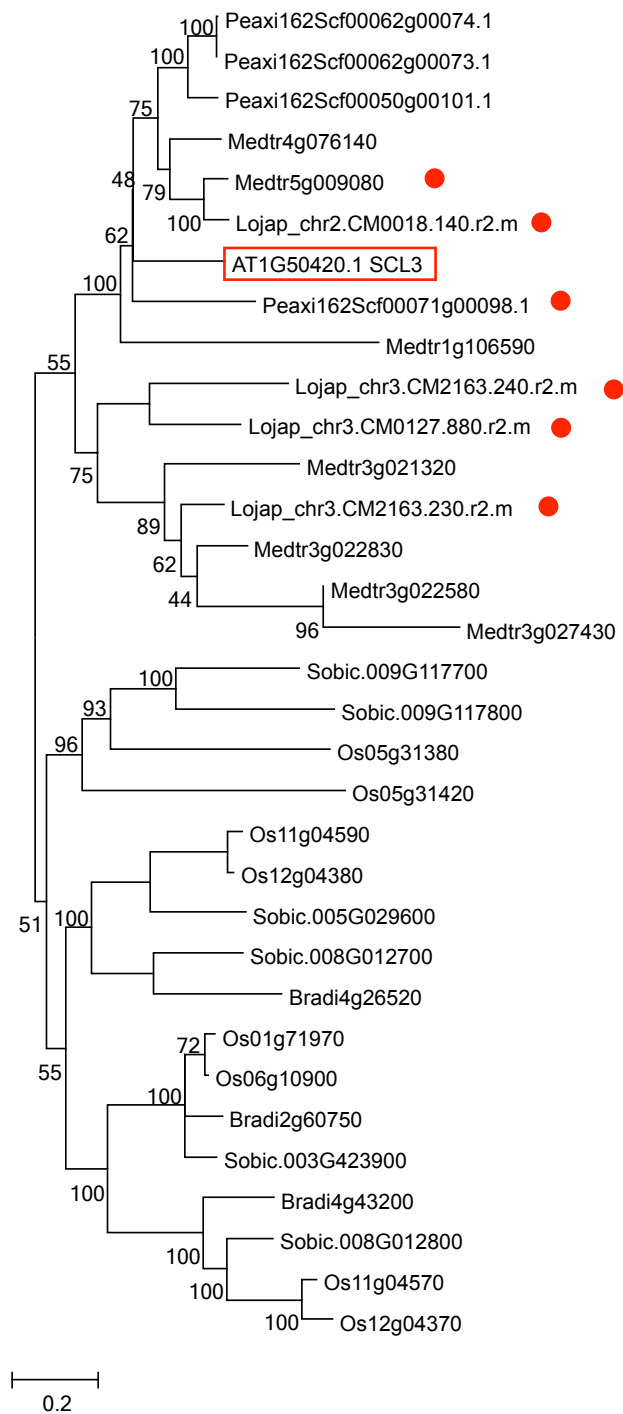

Supplement: Supplementary file 13 — Figure of phylogenetic tree of GRAS proteins in the SCARECROW-LIKE3 subfamily. AM-induced genes from P. axillaris (Peaxi), M. truncatula (Medtr), and L. japonicus (Lojap) are marked with red circles. The closest homologue in A. thaliana (AT) is highlighted with a red frame. Potri: P. trichocarpa; Sobic: S bicolor; Bradi: B. distachyon; Os: O. sativa). The distance bar indicates substitutions per site. (PDF 62 kb) [file 12864_2017_3988_MOESM13_ESM.pdf]

## Additional File 14. Expression of selected GRAS genes in mycorrhizal roots

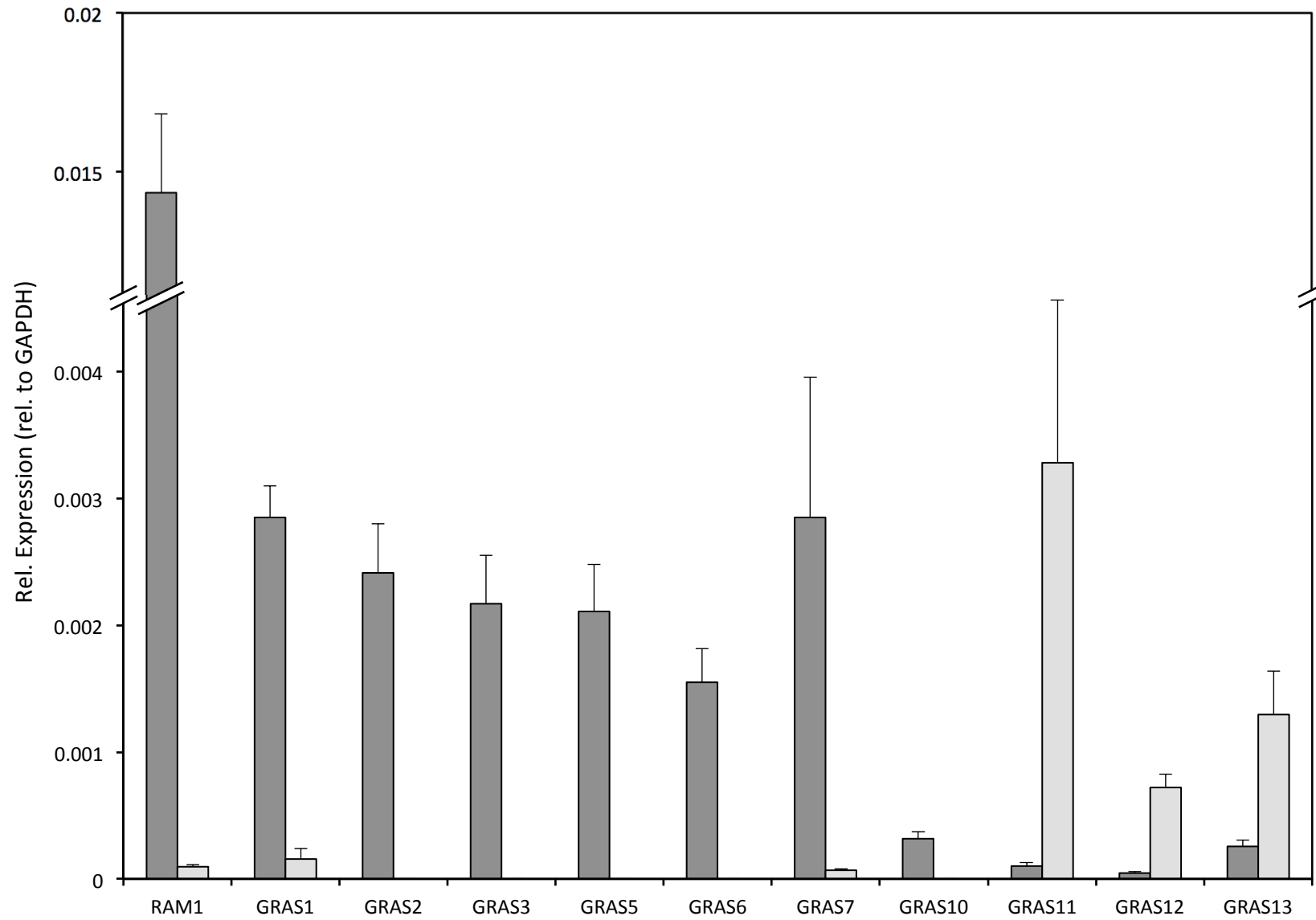

Supplement: Supplementary file 14 — Expression of GRAS genes in mycorrhizal roots. Expression analysis by qPCR of various GRAS transcription factor genes in mycorrhizal roots (dark grey columns) and control roots (light grey columns). In all cases, the expression was significantly different. Identities and gene names of GRAS genes can be found in Additional file 3. (PDF 56 kb) [file 12864_2017_3988_MOESM14_ESM.pdf]

## Additional File 15. Expression of selected GRAS genes in aerial tissues

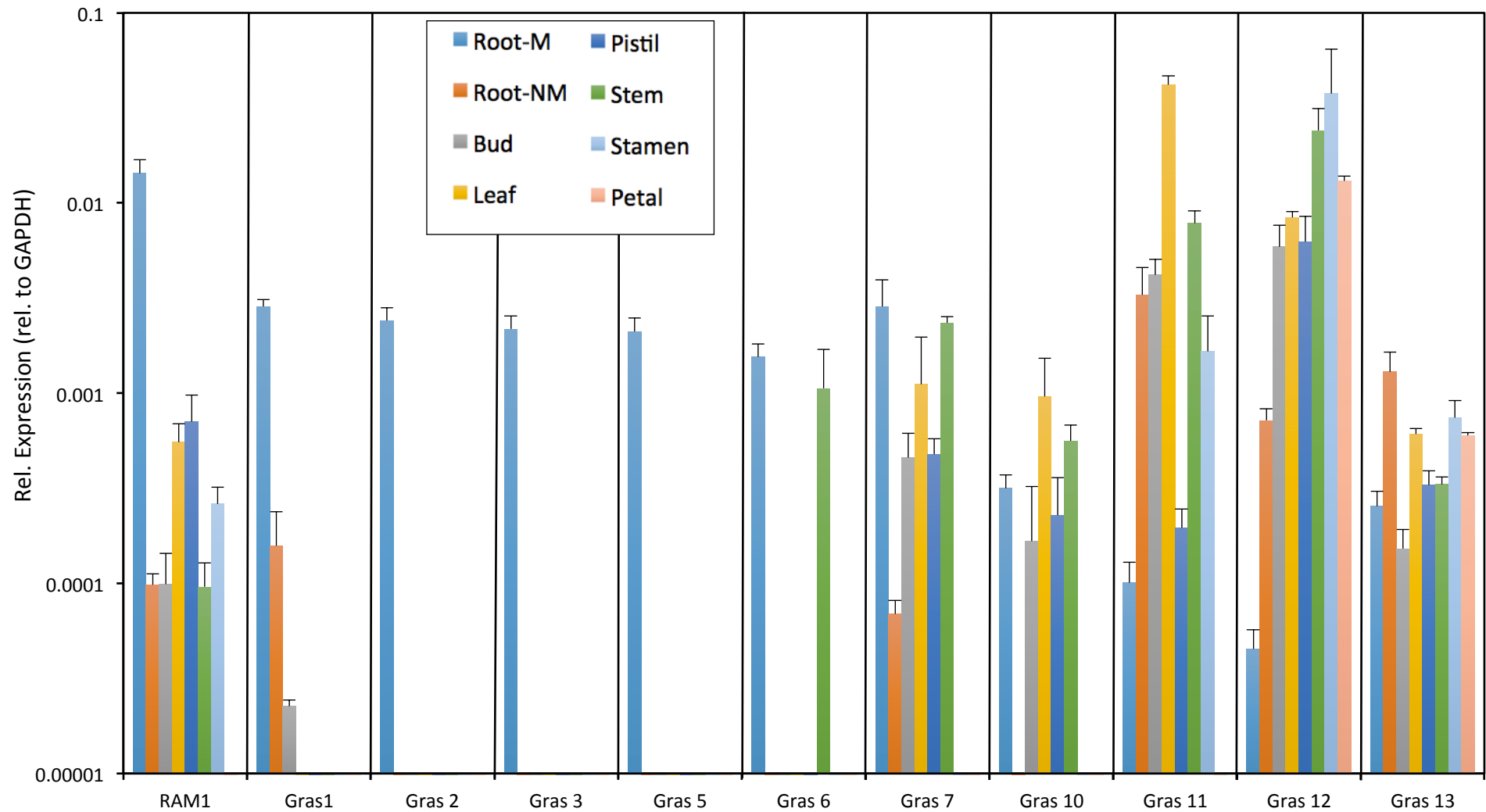

Supplement: Supplementary file 15 — Expression of GRAS genes in various aerial tissues. Global expression analysis by qPCR of various GRAS transcription factor genes in tissues collected from mycorrhizal roots (Root-M), control roots (Root-NM), shoot tips (buds), and various aerial organs (Leaf, Pistil, Stem, Stamen, Petal). Note logarithmic scale of y-axis. Identities and gene names of GRAS genes can be found in Additional file 3. (PDF 96 kb) [file 12864_2017_3988_MOESM15_ESM.pdf]

## Additional File 16. qPCR analysis of selected genes from RNAseq analysis (Fig. 5 - Fig. 8)

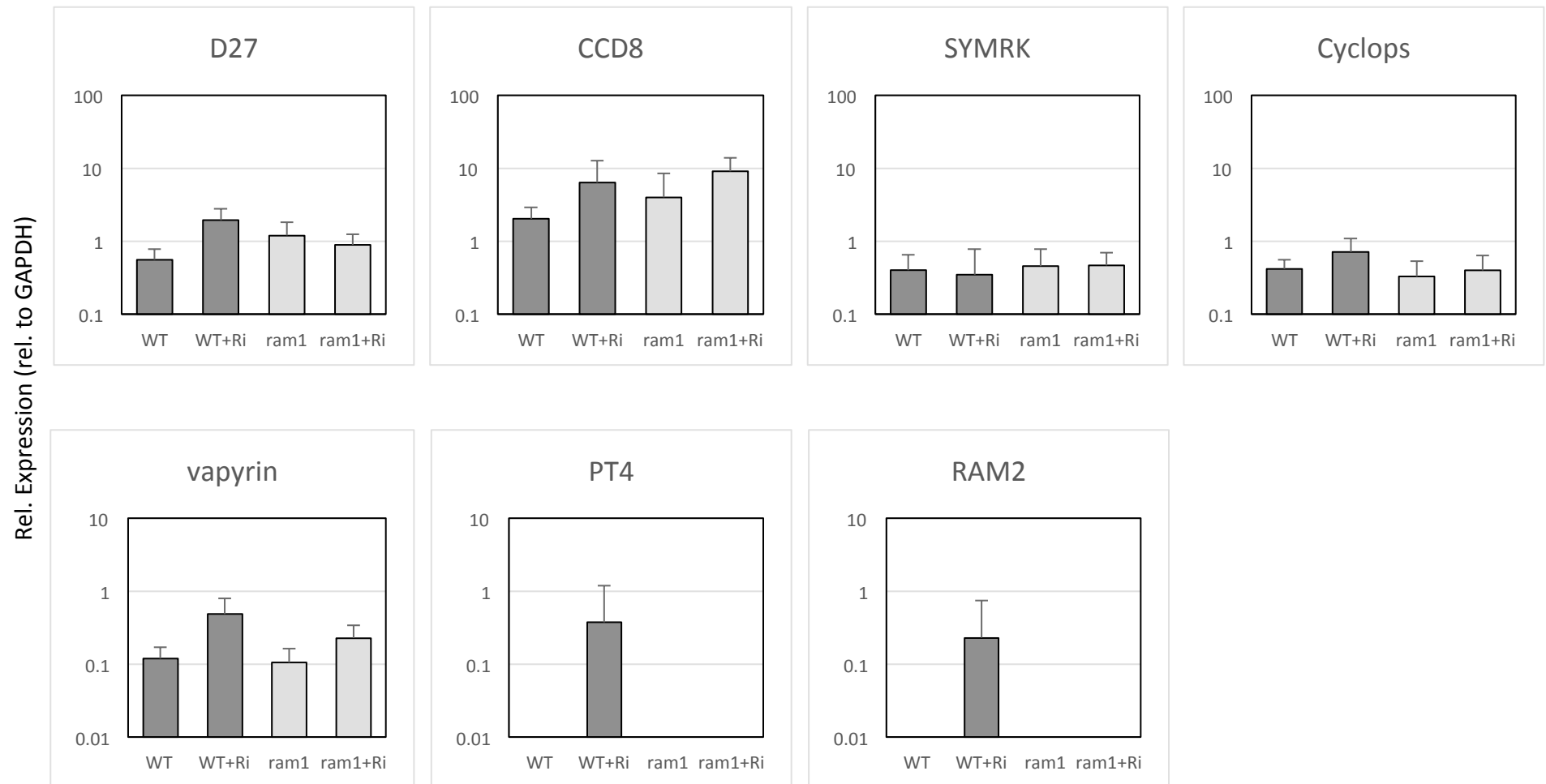

Supplement: Supplementary file 16 — Expression of AM-related genes involved at different stages of AM interaction. Expression analysis by qPCR of D27, CCD8, SYMRK, VAPYRIN, PT4, and RAM2 in wild type (dark grey columns) and ram1 mutants (light grey columns) with the AM fungus R. irregularis (Ri) or in nonmycorrhizal controls. Note logarithmic scale of y-axis. Identities and gene names of GRAS genes can be found in Additional file 3. (PDF 40 kb) [file 12864_2017_3988_MOESM16_ESM.pdf]
