# Supplementary material for: Role of the GRAS transcription factor ATA/RAM1 in the transcriptional reprogramming of arbuscular mycorrhiza in Petunia hybrida
Source: BMC Genomics. 2017 Aug 8;18:589. doi: 10.1186/s12864-017-3988-8 (PMC5549340; doi:10.1186/s12864-017-3988-8)
Supplement: Supplementary file 7 — Table of AM-inducible transcription factors. AM-induced transcription factor genes were classified according to their sequence features. The corresponding induction pattern in Lotus japonicus is given for comparison [47]. (PDF 50 kb) [file 12864_2017_3988_MOESM7_ESM.pdf]

Additional File 7: Transcription factors induced in mycorrhizal roots

	Total	induced in WT	Induced in *ram1*	Xue et al. 2015
**Total**	**1610**	**53**	**17**	**45**
GRAS	78	19	7	18
AP2/ERF	156	9	6	8
MYB	468	4	0	3
NAC	135	4	1	4
MADS	128	3	0	2
C2H2	71	3	2	5
bHLH	182	1	0	NA
B3	82	1	1	NA
bZIP	80	1	0	1
DOF	35	1	0	NA
jumonji	24	1	0	NA
WRKY	86	0	0	1
ARF	25	0	0	1
LOB	60	0	0	1
NIN	NA	NA	NA	1
